# Long-term survival and cure model following liver resection for breast cancer metastases

**DOI:** 10.1007/s10549-018-4714-1

**Published:** 2018-02-20

**Authors:** Aldrick Ruiz, Mylène Sebagh, Dennis A. Wicherts, Carlos Castro-Benitez, Richard van Hillegersberg, Bernard Paule, Denis Castaing, Eric Vibert, Antonio Sa Cunha, Daniel Cherqui, Jean-François Morère, René Adam

**Affiliations:** 1 AP-HP Hôpital Paul Brousse, Centre Hépato-Biliaire, Villejuif, France; 2 Department of Surgery, University Medical Center Utrecht, Utrecht, The Netherlands; 3 Department of Surgery, Academic Medical Center, Amsterdam, The Netherlands; 4 Department of Surgery, Hospital Mexico, San José, Costa Rica; 5 Université Paris-Sud, UMR-S 785, Villejuif, France; 6 Département de Cancérologie, AP-HP Hôpital Paul Brousse, Villejuif, France; 7 Université Paris-Sud, UMR-S 785, 94804 Villejuif, France

**Keywords:** Breast cancer, Liver metastases, Hepatectomy, Cure, And long-term survival

## Abstract

**Introduction:**

Long-term survival is still rarely achieved with current systemic treatment in patients with breast cancer liver metastases (BCLM). Extended survival after hepatectomy was examined in a select group of BCLM patients.

**Patients and methods:**

Hepatectomy for BCLM was performed in 139 consecutive patients between 1985 and 2012. Patients who survived < 5 years were compared to those who survived ≥ 5 years from first diagnosis of hepatic metastases. Predictive factors for survival were analyzed. Statistically cured, defined as those patients who their hazard rate returned to that of the general population, was analyzed.

**Results:**

Of the 139, 43 patients survived ≥ 5 years. Significant differences between patient groups (< 5 vs. ≥ 5 years) were mean time interval between primary tumor and hepatic metastases diagnosis (50 vs. 43 months), mean number of resected tumors (3 vs. 2), positive estrogen receptors (54% vs. 79%), microscopic lymphatic invasion (65% vs. 34%), vascular invasion (63% vs. 37%), hormonal therapy after resection (34% vs. 74%), number of recurrence (40% vs. 65%) and repeat hepatectomy (1% vs. 42%), respectively. The probability of statistical cure was 14% (95% CI 1.4–26.7%) in these patients.

**Conclusions:**

Hepatectomy combined with systemic treatment can provide a chance of long-term survival and even cure in selected patients with BCLM. Microscopic vascular/lymphatic invasion appears to be a novel predictor for long-term survival after hepatectomy for BCLM and should be part of the review when discussing multidisciplinary treatment strategies.

**Electronic supplementary material:**

The online version of this article (10.1007/s10549-018-4714-1) contains supplementary material, which is available to authorized users.

## Introduction

Liver metastases in patients with breast cancer (BCLM) have historically been associated with the worst prognosis compared to other metastatic sites such as the lungs, bone, or brain, with 5-year survival rates of only 4–12% (median survival 4–21 months) [1–3]. Breast cancer in general is a major problem of public health for women worldwide with an estimated 1.7 million women diagnosed with breast cancer in 2012 [4]. A significant proportion of these patients (around 30%) will eventually develop metastatic disease (stage IV). Although systemic treatment for metastatic breast cancer has significantly improved in recent years, dissemination is still associated with poor survival.

Within current guidelines, patients with stage IV breast cancer are only eligible for palliative systemic treatment. The U.S. National Cancer Institute, among other influential organizations, does not mention liver resection as an option for metastatic breast cancer to the liver.

Considering the poor results achieved by current guidelines, the concept of oligimetastatic resection and the existence of unreachable tumor cells deep within tumors (by systemic agents) has become the driving force behind the advocates for resection of limited metastatic disease, especially if they are reactive to systemic treatment [5–7]. Patients with colorectal liver metastases, for example who undergo curative liver resection have seen remarkable results of 5-year survival rates between 30 and 40% and even 50% in selected cases with low surgical mortality or morbidity, something unthinkable for breast cancer liver metastases [8]. So far, only few small retrospective series have been reported regarding the resection of BCLM and there have been no randomized control trials [9–25].

At our institution, our highly selected patients with BCLM have routinely undergone surgical resection since 1985 with previously reported promising results [9]. In general, patients with limited disease experienced more favorable outcome after surgery compared to patients with more extensive tumor involvement. The real potential of prolonged survival or even “cure” in selected patients after hepatic resection is, however, still questioned and liver resection is still not offered as part of advanced breast cancer treatment strategy.

The purpose of this study was to analyze possible indications for and against liver resection in women with BCLM. Furthermore, to study the possibility of exceptional long-term survival, we focused on patients who underwent liver surgery combined with systemic treatment and survived less than 5 year and more than 5 years after liver metastases diagnosis (note: not time of liver resection) with survival beyond 5 years a rarity with current palliative guidelines. In addition, predictive factors and the possibility of “cure” were analyzed.

## Patients and methods

### Study population

All consecutive patients with BCLM, who underwent a partial hepatectomy at our center between January 1985 and December 2012, were selected. Patients were selected from our prospectively maintained institutional database, and each medical record was reviewed to update clinical and pathological data. Additional immunohistochemistry analysis was conducted in those patients with missing information and available tumor tissue. To compare with historical published studies, that reported on patients who were not operated, the starting point was defined as the moment of diagnosis of liver metastases. Patients who survived < 5 years were compared to those who survived > 5 years after first diagnosis of liver metastases.

### Preoperative workup

To be considered for hepatic resection, all patients were required to have received stage-appropriate therapy for their primary tumor. Selection criteria for liver resection were previously presented [9]. In summary, liver resection was proposed to all patients with metastases confined to the liver (or associated to very limited and stable extrahepatic disease), provided that the tumor was controlled by systemic treatment and could be completely resected with a functional remnant liver of at least 30% of the total liver volume. Preoperatively, each patient underwent abdominal ultrasonography and abdominal and thoracic computed tomography (CT), as well as a bone radionuclide scan, to determine the extent of intra- and extrahepatic disease. In the more recent patients, MRI and FDG-PET were more routinely performed to better assess the extent of intra- and extrahepatic tumor spread.

Patients with single and easily resectable liver metastases underwent early surgery without chemotherapy in case of a prolonged disease-free interval (more than 6 months). Patients with large or multiple metastases and a short disease-free interval received preoperative chemotherapy for 2–3 months. Preoperative chemotherapy was furthermore routinely indicated for patients with concomitant extrahepatic disease. The aim of preoperative chemotherapy was to limit tumor spread, to reduce tumor volume, and to exclude patients with rapidly progressive metastatic disease in whom liver resection was unlikely to provide any survival benefit. Chemotherapy consisted of a combination of the classical cytostatic drugs (such as anthracyclines, pyrimidine analogs and taxanes), hormonal therapy (aromatase inhibitors and anti-estrogen agents) or targeted therapy (monoclonal antibodies) to achieve maximum response.

The decision for hepatectomy was taken in a multidisciplinary meeting including surgeons, medical oncologists and radiologists, when the overall surgical strategy could achieve complete tumor resection and the disease was controlled by chemotherapy.

### Hepatic resection

During surgery, abdominal exploration and liver ultrasonography were used to confirm tumor resectability and to evaluate the presence of extrahepatic disease. Parenchymal dissection was done using the ultrasonic dissector (Cavitron Ultrasonic Aspirator, Valleylab, Boulder, CO, USA) and a fenestrated bipolar forceps. The extent of hepatic resection was classified as minor (< 3 hepatic segments) or major (≥ 3 hepatic segments) according to Couinaud’s classification [26]. Clamping of the hepatic pedicle was used if needed to control intraoperative blood loss. Tumor-free resection margins were the objective in all cases and when needed radiofrequency ablation or cryoablation was performed in combination with liver resection to achieve potentially curative surgery. Suspicious lymph nodes on the hepatic pedicle (regional) were resected for pathological review when detected, as were lymph nodes of the celiac trunk or the superior mesenteric artery (distant). However, limited extrahepatic disease was not a contraindication for hepatic resection.

### Postoperative outcome and follow-up

Postoperative mortality was defined as death within the first 60 days following surgery. Postoperative morbidity was defined as any postoperative adverse event, which occurred during the same period. Postoperative complications were divided into hepatic complications, which occurred within the field of liver resection (e.g., biliary fistula), and general complications, which occurred distant from the hepatic resection field (e.g., pneumonia).

All patients were regularly followed at our outpatient clinic, starting 1 month after surgery, then every 4 months for the first 2 years and every 6 months after 2 years. Follow-up consisted of a history, physical examination and radiological imaging. Abdominal ultrasound and abdominal and thoracic CT imaging were alternately performed.

### Statistical analysis

Median follow-up time for the whole population was well beyond 5 years (108 months). As 5-year survival could be considered as a valuable turning point for the evaluation of outcome, the whole series was divided into two groups; patients who survived < 5 years versus patients who survived ≥ 5 years after first diagnosis of liver metastases. Categorical variables were compared between groups by the Chi square (*χ*^2^) test and continuous variables were compared using the independent-sample *t* test. Overall survival probabilities were estimated using the Kaplan–Meier method and were compared using the log-rank test. Univariate analysis was performed to determine factors related to a survival of patients who survived beyond 5 years by using the log-rank test. To identify independent predictors of long-term survival, all factors with an univariate significance of *P* < 0.10 were entered into a Cox proportional hazard model. To correct for missing values, multiple imputations were performed twenty times and pooled. Regressions are presented as original and imputed. All statistical analyses were performed with SPSS version 21.0 (SPSS Inc., Chicago, IL, USA), and statistical significance was determined at *P* ≤ 0.05.

### Cure model

When a patient’s observed hazard rate returns to that of the general population, that patient may be considered cured of the disease, because the risk of death is just as likely as for any member of the general population. [27] The estimation of expected survival and of expected hazard of the general population was derived from population-based survival tables obtained from the French National institute of Statistics and Economics, matched by age [28]. Survival time in our study group was defined as the period between hepatic resection (intervention to achieve cure) and date of death or last follow-up. The potential of cure was calculated using STATA software (StataCorp. 2011, College Station, TX: StataCorp LP) as described in several published works [27, 29–31].

## Results

### Study population

Between January 1985 and December 2012, 139 consecutive female patients underwent 162 hepatectomies for BCLM at our institution. Of these 139 patients, 120 (86%) underwent a single hepatectomy and 19 (14%) underwent a second hepatectomy. In 4 (3%) patients, a third hepatectomy was needed.

In total, 43 (31%) patients lived ≥ 5 years after first diagnosis of liver metastases. These 43 patients were compared to 96 (69%) patients who survived < 5 years after liver metastases diagnosis regardless of the number of hepatectomies.

### Long-term overall and disease-free survival

**Fig. 1:**
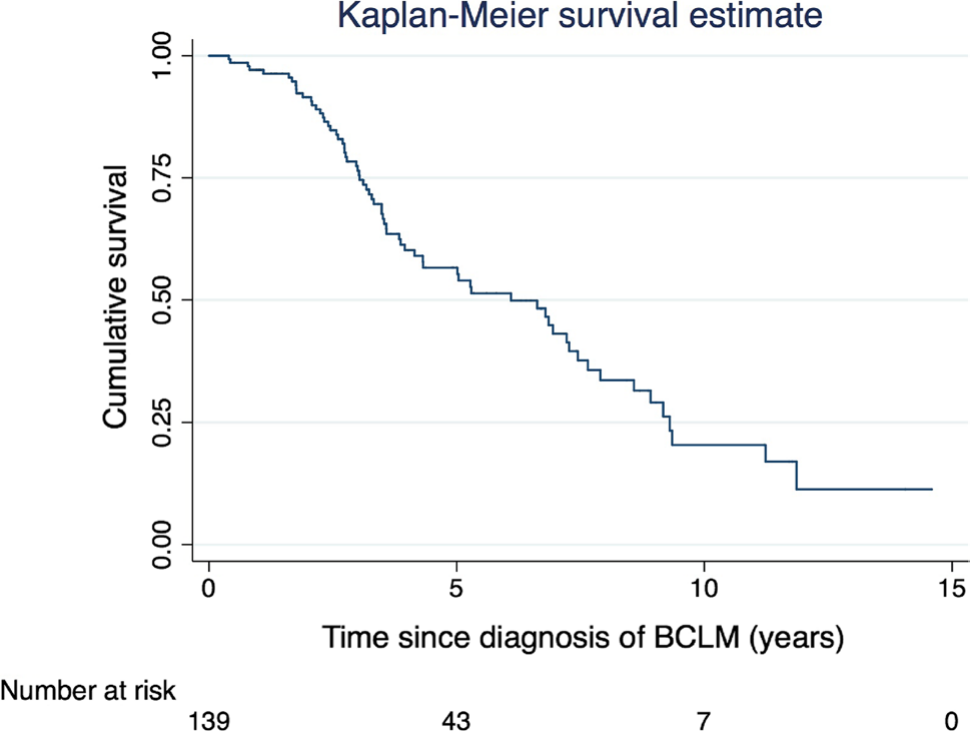
Survival estimates

Median follow-up time was 108 months for the whole series. The 7- and 10-year overall survival in the ≥ 5 years group was 76% and 36%, respectively. Seven of the 43 patients (16%) are alive more than 10 years since liver metastases diagnosis (5% of the total population) with a longest survival of almost 15 years (175 months) (Figure 1) . Of the 43 patients who survived ≥ 5 years, 22 (51%) had no hepatic recurrence at last follow-up. Of the 96 patients that survived < 5 years, 58 patients (60%) had no hepatic recurrence at last follow-up.

Median disease-free survival for the whole series was 33 months. Median disease-free survival was 25 and 43 months in the < 5 and ≥ 5 years groups, respectively (Supplemental Table 1).

### Patient and tumor characteristics comparison

Patient and tumor characteristics of both groups are summarized in Table 1. Patients who survived ≥ 5 years had a shorter time interval between primary tumor and liver metastases diagnosis (43 ± 29 months vs. 50 ± 47 months, *P* = 0.026). The proportion of patients with concomitant extrahepatic disease at first hepatectomy was higher in patients who survived < 5 years (34% vs. 19%, *P* = 0.071).

**Table 1:** Patient and tumor characteristics comparison *n* = 139

	< 5-years group	%	≥ 5-years group	%	*p*
Primary breast tumor^§^					
Adenocarcinoma					
Ductal	51	86	26	93	0.490
Lobular	8	14	2	7	
Differentiation					
Well	3	5	1	3	0.503
Moderate	34	61	22	73	
Poor	19	34	7	23	
Surgical removal					
Breast conserving	42	45	25	60	0.140
Mastectomy	51	55	17	40	
Receptor status					
ER positive					
No	9	20	3	14	0.737
Yes	36	80	19	86	
PR positive					
No	15	35	7	35	1.000
Yes	28	65	13	65	
Her2/Neu positive					
No	24	69	9	60	0.746
Yes	11	31	6	40	
ER/PR negative					
No	36	84	18	90	0.706
Yes	7	16	2	10	
Systemic treatment					
Neoadjuvant chemo					
No	93	97	41	95	0.645
Yes	3	3	2	5	
Adjuvant chemo					
No	39	41	19	44	0.713
Yes	57	59	24	56	
Post op hormonal therapy					
No	66	69	26	61	0.341
Yes	30	31	17	39	
Post op radiotherapy					
No	32	33	13	30	0.845
Yes	64	67	30	70	
Breast cancer liver metastases					
Sync					
Synchronous	7	7	4	10	0.738
Metachronous	87	93	38	90	
Mean interval months between primary and BCLM ± SD	50 ± 47 months		43 ± 29 months		0.026*
Mean number of BCLM ± SD	2 ± 2		2 ± 2		0.803
Mean maximum tumor size ± SD, mm	33 ± 17		37 ± 20		0.312
Distribution					
Bilateral	29	32	17	44	0.232
Unilateral	62	68	22	56	
Concomitant extra-hepatic disease					
No	63	66	35	81	0.071
Yes	33	34	8	19	
Preoperative chemotherapy					
No	30	31	11	26	0.551
Yes	66	69	32	74	
Hepatectomy					
Mean age at hepatectomy ± SD	53 ± 11 years		48 ± 10 years		0.208
Timing of hepatectomy					
Year < 2000	36	38	17	40	0.852
Year > 2000	60	62	26	60	
Extent of resection					
Limited resection (< 3 segments)	38	40	19	44	0.710
Major resection (≥ 3 segments)	58	60	24	56	
Type of resection					
Anatomical	32	33	17	40	0.657
Wedge	35	37	13	30	
Anatomical+wedge	29	30	13	30	
Histopathology					
Mean number of resected metastases ± SD	3 ± 2		2 ± 2		0.023*
Solitary tumor					
No	57	63	19	46	0.090
Yes	34	37	22	54	
Mean maximum size ± SD, mm (M)	30 ± 26		26 ± 16		0.374
Resection margin					
R0	52	58	25	66	0.435
R+	38	42	13	34	
Hormonal receptor status					
ER−	40	46	7	21	0.021
ER+	48	54	26	79	
PR−	57	65	24	73	0.516
PR+	31	35	9	27	
Her2/neu−	60	70	23	72	1.000
Her2/neu+	26	30	9	28	
Triple negative (ER, PR, HER2/NEU)					
No	66	75	28	85	0.329
Yes	22	25	5	15	
Lymphatic embolus					
No	28	35	21	66	0.003*
Yes	53	65	11	34	
Vascular embolus					
No	33	37	24	63	0.011*
Yes	56	63	14	37	
Regional lymph node invasion					
Negative for tumor cells	9	41	4	57	0.667
Positive for tumor cells	13	59	3	43	
Distant lymph node invasion					
Negative for tumor cells	12	75	1	100	1.000
Positive for tumor cells	4	25	0	0	
Post hepatectomy					
Postoperative chemotherapy					
No	35	37	9	21	0.078
Yes	61	64	34	79	
Hormonal therapy after resection					
No	63	66	11	26	0.000*
Yes	33	34	32	74	
Monoclonal therapy after resection					
No	72	75	25	58	0.071
Yes	24	25	18	42	
Recurrence					
No	58	60	15	35	0.006*
Yes	38	40	28	65	
Repeat hepatectomy					
No	95	99	25	58	0.000*
Yes	1	1	18	42	

^§^Referring center for hepatectomy, primary usually is treated in a different hospital, *p-value < 0.05

The mean number of tumors resected in the ≥ 5 years group was 2 compared to 3 in < 5 years group (*P* = 0.023). More patients in the ≥ 5 years group had a solitary liver metastasis compared to the < 5 years group (54% vs. 37%, *P* = 0.090). Estrogen receptor positive tumors were more prevalent in the ≥ 5 years group (79% vs. 54%, *P* = 0.021). The proportion of patients with microscopic lymphatic and vascular invasion was higher in patients who survived < 5 years (lymphatic; 65% vs. 34%, *P* = 0.003, vascular; 63% vs. 37%, *P* = 0.011). Patients who lived ≥ 5 years more frequently received hormonal therapy after hepatic resection (74% vs. 34%, *P* < 0.000).

After hepatic resection, the proportion of hepatic recurrences was higher in the ≥ 5 years group (65% vs. 40% (*P* = *0.006*). Repeat hepatectomy was performed in 1 of the patients who survived < 5 years and in 18 patients who survived ≥ 5 years (1% vs. 42%, *P* < 0.000).

#### Short-term outcome

There were more general complications in the < 5 years group than in the ≥ 5 years group (25% vs. 10%, *P* = 0.061). The mean hospital stay was similar in both groups; 11 vs. 10 days (*P* = 0.420). The 60-day mortality was 2% after hepatic resection for the whole series (Table 2).

**Table 2:** Comorbidity

	< 5-year group	%	≥ 5-year group	%	*p*
Morbidity^a^					
No	63	66	28	72	0.684
Yes	32	34	11	28	
General complications					
No	71	75	35	90	0.062
Yes	24	25	4	10	
Hepatic complications					
No	83	87	32	82	0.425
Yes	12	13	7	18	
Biliary leakage	2	17	4	57	
Biliary leakage+infected collection	1	8	1	14	
Biliary leakage+noninfected collection	1	8	0	0	
Hemorrhage	0	0	1	14	
Infected collection	4	33	0	0	
Noninfected collection	3	25	1	14	
Mean hospital stay, days ± SD (M)	11 + 7		10 + 4		0.420

*n* = 139

^a^General and/or hepatic complication

### Predictive factors of long-term survival

At univariate analysis, factors significantly related to better survival in those patient who lived longer than 5 years were: time interval between primary tumor and hepatic metastases equal or more than 18 months, resected metastasis size smaller than 35 mm, absence of microscopic vascular invasion or combination of vascular and lymphatic invasion, absence of chemotherapy after hepatic resection (Table 3).

**Table 3:** Univariate and multivariate analysis of overall survival in patients that survived 5 years or longer after hepatectomy since date of diagnosis

	*n*	%	7 years (%)	10 years (%)	Median (Mo)	Log rank	*P* ^d^	*P* ^e^	Hazard ratio	(95% CI)	
Liver metastases											
Time of appearance											
Synchronous	4	9	0	0	–	0.462					
Metachronous	38	88	74	37	–						
Interval primary tumor and metastasis											
< 18 months	6	14	0	0	82	0.01	0.035	NS			
≥ 18 months	35	81	81	40	111						
Tumor number											
Solitaire	20	47	81	52	142	0.157					
> 1	23	53	72	21	110						
Maximal tumor size											
< 30 mm	14	33	93	0	94	0.680					
≥ 30 mm	23	53	76	50	111						
Distribution											
Unilateral	22	51	84	52	134	0.187					
Bilateral	17	40	61	12	107						
Segments involved											
1	18	42	79	47	102	0.999					
> 1	20	47	79	36	110						
Chemo tx pre hepatectomy^a^											
No	11	26	80	48	94	0.430					
Yes	32	74	75	30	110						
Hormone tx pre hepatectomy^b^											
No	36	84	71	32	102	0.108					
Yes	7	16	100	50	107						
Targeted tx pre hepatectomy^c^											
No	36	84	76	36	110	0.985					
Yes	7	16	75	0	86						
Extra hepatic metastases											
No	35	81	77	37	110	0.644					
Yes	8	19	73	29	91						
Sites of Extrahepatic disease											
Brain	1	2									
Bone	2	5									
Lung	4	9									
Lymph Node	1	2									
First hepatectomy											
Age (E3)											
< 50 years.	26	60	77	29	102	0.613					
≥ 50 years	17	40	75	44	111						
Hepatic resection (E3)											
Minor (< 3)	17	40	81	37	111	0.978					
Major (≥ 3)	26	60	72	33	120						
Type of resection (E3)											
Anatomical	19	44	88	62	134	0.287					
Both	24	56	66	23	107						
Not Anatomical											
Tumor number (E3)	17	40	74	41	110	0.900					
Solitaire	13	30	69	46	91						
> 1	13	30	100	21	111						
Maximal tumor size (E3)											
< 35 mm	26	60	74	19	102	0.033	NS	NS			
≥ 35 mm	12	28	92	69	142						
Resection margin (E3)											
R0	25	58	82	41	111	0.209					
R1	13	30	69	19	91						
R2	0	0	–	–	–						
Hormone receptor status (E3)											
ER−	7	16	86	86	(124)	0.713					
ER+	26	60	83	30	110						
No tumor cells	4	9	67	0	(98)						
PR−	24	56	76	33	107	0.543					
PR+	9	21	100	37	112						
No tumor cells	4	9	67	0	(98)						
HER2−	23	53	76	37	102	0.465					
HER2+	9	21	100	42	111						
No tumor cells	4	9	67	0	(98)						
Double neg (ER−, PR−) (E3)											
No	8	19	100	23	110	0.877					
Yes	25	58	77	39	107						
No tumor cells	4	9	67	0	(98)						
Triple neg (ER−, PR−, HER2−) (E3)											
No	28	65	84	34	110	0.874					
Yes	5	12	80	0	(81)						
No tumor cells	4	9	67	0	(98)						
Vascular invasion (E3)											
No	24	56	92	47	111	0.06	NS	NS			
Yes	14	33	64	13	91						
Lymphatic Invasion (E3)											
No	21	49	95	50	111	0.149					
Yes	11	26	80	15	102						
Vascular and/or lymphatic Invasion (E3)											
No	19	44	95	54	134	0.078	NS	0.023	3.485	1.184	10.250
Yes	19	44	70	20	91						
Lymph Node Invasion (E3)											
No	4	9	75	50	87	0.222					
Yes	3	7	0	0	79						
Chemo tx post hepatectomy^a^ (E3−>)											
No	9	21	88	73	142	0.077	NS	NS			
Yes	34	79	73	22	107						
Hormone tx post hepatectomy^b^ (E3−>)											
No	11	26	89	64	142	0.088	0.007	0.023	4.197	1.217	14.471
Yes	32	74	72	24	107						
Targeted tx post hepatectomy^c^ (E3−>)											
No	25	58	83	46	111	0.209					
Yes	18	42	64	0	110						
Radiofrequency ablation, cryo ablation or arterial embolization (E3)											
No	38	88	79	41	110	0.159					
Yes	5	12	40	0	82						
Post hepatectomy course											
Chemo tx peri hepatectomy^a^ (E2− > E3−>)											
No	0	0	100	50	94	0.802					
Yes	40	93	74	34	110						
Hormone tx peri hepatectomy^b^ (E2− > E3− >)											
No	9	21	88	63	142	0.137					
Yes	34	79	73	25	107						
Targeted tx peri hepatectomy^c^ (E2− > E3−>)											
No	23	53	87	48	111	0.110					
Yes	20	47	59	0	110						
Hepatic Recurrence (E2.2)											
No	15	35	86	53	(116)	0.216					
Yes	28	65	71	29	102						
Interval first hepatectomy to recurence (E3− > E2.2)											
< 12 months	6	14	60	40	86	0.670					
≥ 12 months	12	28	73	26	110						
Tumor number											
Solitair	10	23	77	43	111	0.481					
> 1	17	40	71	21	102						
Repeat hepatectomy											
No	10	23	64	0	87	0.650					
Yes	18	42	74	47	112						

*n* = 43

*E1* primary tumor, *E2* diagnosis hepatic metastases, *E3* hepatectomy, *E4* extra hepatic metastases, *E2.2* hepatic recurrence

^a^Antracyclines, pyrimidine, taxanes, platinum, vinca; single or in combinations

^b^Aromatase inhibitor and anti-estrogen

^c^Monoclonal antibodies, () = estimated mean+ = in ER+ or PR+ patients

^d^Multivariate

^e^multivariate with 20 × imputation of missing values, *tx* therapy, *NS* not significant *p* > 0.05

Multivariate analysis identified two factors associated with long-term survival in patients who lived ≥ 5 years. Patients with microscopic vascular and/or lymphatic invasion had a three and half fold chances of dying compared to patients with no vascular or lymphatic invasion (7-year survival 70% vs. 95%, respectively, HR 3.485, range 1.184-10.250, *P* = 0.023). Patients who received hormonal therapy after hepatic resection had a four fold chance of dying compared to those that did not receive hormonal therapy (7-year survival 72% vs. 89%, respectively, HR 4.197, range 1.217–14.471, *P* = 0.023).

### Probability of cure

**Fig. 2:**
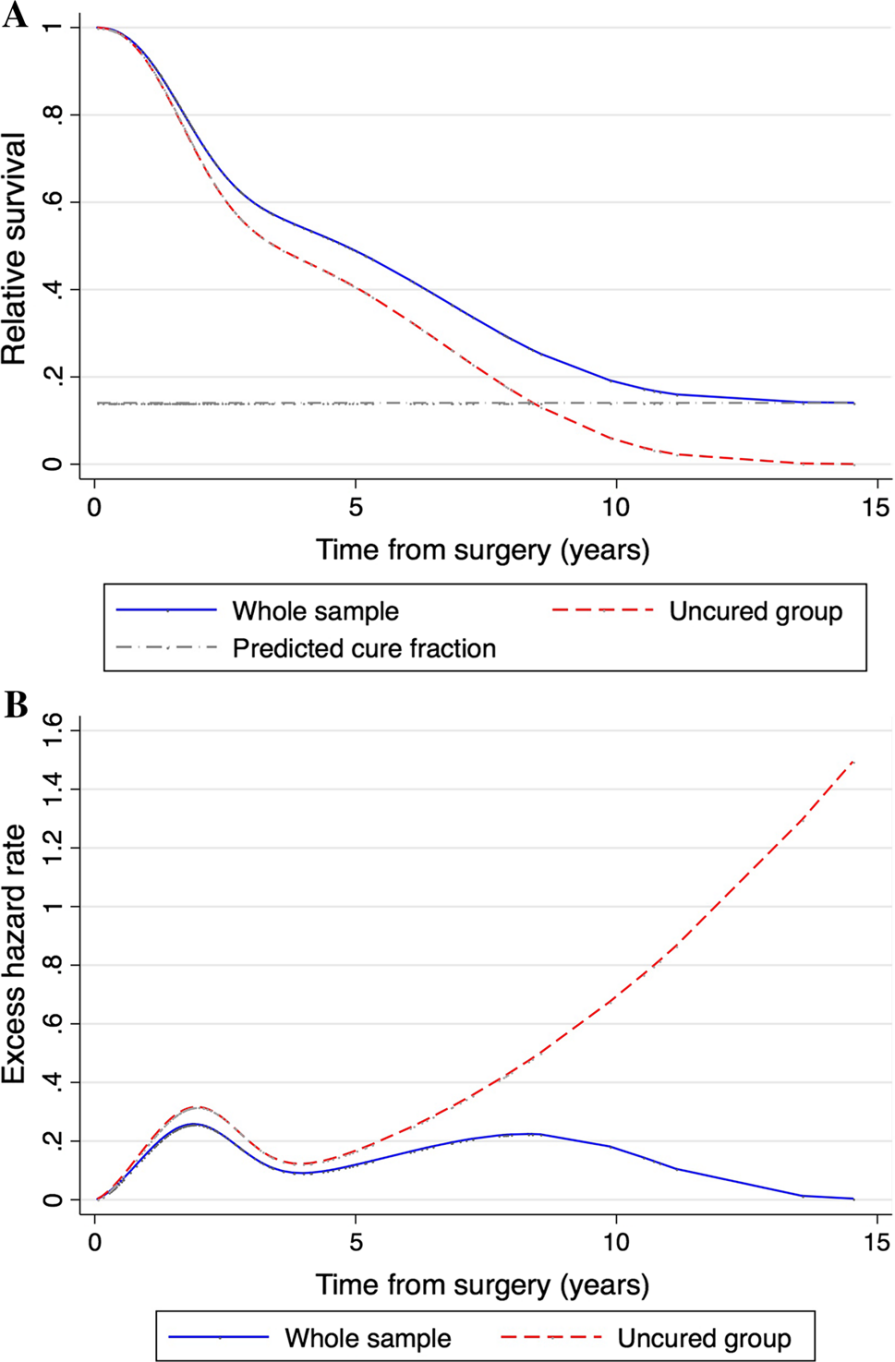
Cure model results. **a** Relative survival of the entire group of patients and the uncured patients. **b** Excess hazard rate of the entire study group and the uncured patients

In the entire study population, the probability of being cured of BCLM by hepatic resection combined with systemic treatment was 14% (95% CI 1.4–26.7%) (Fig. 2a). The excess of hazard after surgery started from a 2% increased risk of death early after surgical resection with respect to the general population (Fig. 2b). In the first two postoperative years, the excess hazard increased to approximately 25.8% in the entire group and was up to 32.9% in non-cured patients. After a parallel trajectory of risk of death up to 8.3 years, the entire group demonstrated a progressive reduction in the hazard while the hazard for non-cured patients progressively increased after the 4th year after surgery. The excess of hazard in the entire group decreased towards the general population hazard at 13.6 years after hepatic resection, indicating that after this time point, a patient still alive could be considered cured with 99% certainty.

## Discussion

Long-term survival of BCLM is almost never achieved when liver metastases remain unresected [32–37]. Following hepatectomy, the 3- and 5-year survival rates were 58 and 47%, respectively, for the whole series. Of the 43 patients who survived beyond 5 years, 76% survived 7 years and even 35% survived 10 years after the time of first diagnosis. These results support the indication of hepatic resection for BCLM in selected patients and suggest that some of these patients can even be cured. This strategy also relates with current developments in local treatment of oligometastasis in other cancers such as colorectal carcinomas as part of a more individual tailored approach. The value of liver resection in these patients is also reflected in the fact that even though a portion of patients developed hepatic recurrences, long-term survival can still be achieved when repeat hepatectomy is performed.

Although systemic treatment of breast cancer patients has developed in the past decades, survival of patients with BCLM is still poor with 5-year survival rates of only 4–12% when applying current guidelines. Resection of BCLM remains controversial and is not generally accepted. Few articles have been published describing the possible benefit of surgery in mostly small study populations varying from 2 to 115 patients with a 5-year survival rate ranging from 27 to 50%.

Our series, representing a small proportion of the total number of patients with stage IV breast cancer, is without a doubt a selected group of patients. However, these results have never been expected before, based on the historical reported survival of breast cancer patients with hepatic metastases. This series is the only series to date focusing on the possibility of real long-term survival of BCLM after hepatic resection in an experienced hepatobiliary center. Two different approaches were implemented to highlight key factor that might help selecting patients with better chances of survival and a statistical cure model was constructed that compares the hazard rate of the general population to that of this series over time.

Predictive factors of ≥ 5 year survival were: interval more than 18 months between primary breast tumor and diagnosis of hepatic metastases, size of resected metastases < 35 mm, absence of microscopic vascular and lymphatic invasion, absence of hormonal therapy after resection and repeat hepatectomy in case of tumor recurrence.

Time interval and tumor size are well-documented predictors for overall survival and are related to tumor biology. In several publications, a time interval of 1–2 years after removal of the primary tumor was reported as a significant factor of survival [38].

Sadot et al. compared 69 operated patients to 98 systemically treated in a single center looking at historically selected patients for treatment groups [39]. Even though they concluded that hepatic resection was not associated with survival advantages (median OS: 50 vs 45 months; 5-year OS: 38% vs 39%), a significant recurrence-free interval was seen.

There is no publication that reported on the survival impact of microscopy invasion into vascular or lymphatic structures. Besides the widely accepted residual classification (R0, R1 and R2) for microscopic invasion into the surgical field, we believe that this characteristic should be part of a standard histological review in order to further explore its utility as possible selection criteria for further intervention.

The negative long-term survival effect of postoperative hormonal treatment (idiopathic menopausal state) has never been presented or investigated given the historically documented marginal survival rate of these patients with conventional systemic treatment only. It is known that (early) menopausal state is related to an increased risk of a variety of diseases including cardiovascular disease and this might explain this finding [40]. Given the rise in acceptance of hepatic resection for breast cancer liver metastases, more investigation in a larger cohort will shed more light into whether the benefit of hormonal therapy outweighs the risks in this subset of patients.

Among the patients that survived beyond 5 years we found a higher rate of recurrences but also a higher rate of repeat hepatectomy. This result confirms the importance of an aggressive approach through multiple hepatectomies when surgically possible. The potential benefit of repeat hepatectomy has been presented in a previous published work [41].

It remains controversial to explore the idea of cure given the complexity of the disease and the notion that total eradication of disseminated cell is very unlikely. A cure model was used to compare the hazard rate of our highly selected group of patients to that of the general population. We found that 19 patients (14%) of the whole series had their chance of death reduced to that of the general population. This outcome has never been reported for breast cancer liver metastases patients treated by systemic treatment alone. This result further strengthens the indication for hepatic resection in patients with BCLM.

Our results might be based on our highly selected population but should not be dismissed as selection bias since these results are rarely achieved by conventional palliative therapy. Our study should serve as a guide to select women who might and might not benefit from an aggressive approach and stimulate future studies.

In conclusion, we believe that hepatectomy for BCLM should be considered in all patients when technically feasible and responding to systemic treatment. The current study shows that hepatectomy provides a chance of extreme long-term survival or even statistical cure in selected patients without increased morbidity. Something that was unthinkable within the current palliative approach to BCLM patients. Accurate selection of patients for hepatectomy remains crucial.

## Available data and material

The datasets used and/or analyzed during the current study are available from the corresponding author on reasonable request.

## Electronic supplementary material

Below is the link to the electronic supplementary material.

Supplementary material 1 (DOCX 106 kb)
