# Supplementary material for: Long-term survival and cure model following liver resection for breast cancer metastases
Source: Breast Cancer Res Treat. 2018 Feb 20;170(1):89–100. doi: 10.1007/s10549-018-4714-1 (PMC5993851; doi:10.1007/s10549-018-4714-1)
Supplement: Supplementary file 1 — Supplementary material 1 (DOCX 106 kb) [file 10549_2018_4714_MOESM1_ESM.docx]

# Suplemental

## Table 1. Univariate and multivariate analysis of disease free survival in patients who survived 5 years or longer after hepatectomy since date of diagnosis. N=43

|  | |  |  |  |  |  |  |  |  |  |  |  |  |  |  |  |  |  |  |  |  |  |
| --- | --- | --- | --- | --- | --- | --- | --- | --- | --- | --- | --- | --- | --- | --- | --- | --- | --- | --- | --- | --- | --- | --- |
| Primary Tumor | | | | *N* | *%* | *3 yr %* | | *5 yr %* | *Median (Mo)* | | Log rank | | *P §* | | *P §§* | | *Hazard Ratio* | | (95% CI) | | | |
| Hormone receptor status | | | |  |  |  | |  |  | |  | |  | |  | |  | |  | |  | |
| PR- | | | | 7 | 16 | 71 | | 71 | (90) | | 0.059 | | NS | | NS | |  | |  | |  | |
| PR+ | | | | 12 | 28 | 42 | | 33 | 25 | |  | |  | |  | |  | |  | |  | |
| Liver metastases | | | | *N* | *%* | *3 yr %* | | *5 yr %* | *Median (Mo)* | | Log rank | | *P §* | | *P §§* | | *Hazard Ratio* | | (95% CI) | | | |
| Interval primary tumor and metastasis | | | |  |  |  | |  |  | |  | |  | |  | |  | |  | |  | |
| < 24 months | | | | 9 | 21 | 33 | | 22 | 16 | | 0.088 | | NS | | NS | |  | |  | |  | |
| ≥ 24 months | | | | 31 | 72 | 56 | | 46 | 45 | |  | |  | |  | |  | |  | |  | |
| First hepatectomy | | | | *N* | *%* | *3 yr %* | | *5 yr %* | *Median (Mo)* | | Log rank | | *P §* | | *P §§* | | *Hazard Ratio* | | (95% CI) | | | |
| Maximal tumor size | | | |  |  |  | |  |  | |  | |  | |  | |  | |  | |  | |
| <20 mm | | | | 17 | 40 | 69 | | 57 | 61 | | 0.057 | | NS | | 0.030 | | 2.818 | | 1.103 | | 7.199 | |
| ≥ 20 mm | | | | 20 | 47 | 40 | | 30 | 25 | |  | |  | |  | |  | |  | |  | |
| Vascular invasion | | | |  |  |  | |  |  | |  | |  | |  | |  | |  | |  | |
| No | | | | 23 | 53 | 73 | | 58 | 63 | | 0.002 | | 0.01 | | 0.027 | | 2.946 | | 1.129 | | 7.683 | |
| Yes | | | | 14 | 33 | 21 | | 14 | 20 | |  | |  | |  | |  | |  | |  | |
| Chemo tx post hepatectomy * | | | |  |  |  | |  |  | |  | |  | |  | |  | |  | |  | |
| No | | | | 9 | 21 | 100 | | 75 | (111) | | 0.005 | | NS | | NS | |  | |  | |  | |
| Yes | | | | 33 | 77 | 41 | | 32 | 32 | |  | |  | |  | |  | |  | |  | |
| Hormone tx post hepatectomy ** | | | |  |  |  | |  |  | |  | |  | |  | |  | |  | |  | |
| No | | | | 10 | 23 | 79 | | 56 | (90) | | 0.086 | | NS | | NS | |  | |  | |  | |
| Yes | | | | 32 | 74 | 46 | | 36 | 33 | |  | |  | |  | |  | |  | |  | |
| Targeted tx post hepatectomy *** | | | |  |  |  | |  |  | |  | |  | |  | |  | |  | |  | |
| No | | | | 25 | 58 | 75 | | 62 | 103 | | 0.000 | | 0.015 | | 0.002 | | 3.998 | | 1.66 | | 9.63 | |
| Yes | | | | 17 | 40 | 24 | | 12 | 24 | |  | |  | |  | |  | |  | |  | |
| Radiofrequency ablation, cryoablation or arterial embolization | | | |  |  |  | |  |  | |  | |  | |  | |  | |  | |  | |
| No | | | | 37 | 86 | 61 | | 46 | 48 | | 0.000 | | 0.014 | | 0.026 | | 4.409 | | 1.199 | | 16.221 | |
| Yes | | | | 5 | 12 | 0 | | 0 | 15 | |  | |  | |  | |  | |  | |  | |
| Post hepatectomy course | | | | *N* | *%* | *3 yr %* | | *5 yr %* | *Median (Mo)* | | Log rank | | *P §* | | *P §§* | | *Hazard Ratio* | | (95% CI) | | | |
| Targeted tx peri hepatectomy *** | | | |  |  | |  |  | |  | |  | |  | |  | |  | |  | |  |
| No | | | | 23 | 53 | 73 | | 60 | 103 | | 0.001 | | NS | | *NS* | |  | |  | |  | |
| Yes | | | | 19 | 44 | 29 | | 17 | 28 | |  | |  | |  | |  | |  | |  | |
| *Targeted tx post hepatectomy ***+* | | | |  |  | |  |  | |  | |  | |  | |  | |  | |  | |  |
| No | | | | *5* | *56* | *60* | | *60* | *(89)* | | *0.084* | | *NS* | | *NS* | |  | |  | |  | |
| Yes | | | | *4* | *44* | *25* | | *25* | *14* | |  | |  | |  | |  | |  | |  | |
| * Antracyclines, pyrimidine, taxanes, platinum, vinca; single or in combinations | | | | | | | | | | | | | | | | | | | | | | |
| ** Aromatase inhibitor and anti estrogen, *** Monoclonal antibodies, ()= estimated mean, + = in ER+ or PR+ patients | | | | | | | | | | | | | | | | | | | | | | |
| § Multivariate, §§ multivariate with 20x imputation of missing values, tx= therapy, NS= not significant p>0.05 | | | | | | | | | | | | | | | | | | | | | | |
